# Seeing is believing: the Bicoid protein reveals its path

**DOI:** 10.1186/s41065-018-0067-3

**Published:** 2018-09-11

**Authors:** Stefan Baumgartner

**Affiliations:** Department of Experimental Medical Sciences, Lund University, BMC D10, S-22184 Lund, Sweden

**Keywords:** *Bicoid*, Gradient, mRNA, ARTS, SDD

## Abstract

In this commentary, I will review the latest findings on the Bicoid (Bcd) morphogen in *Drosophila*, a paradigm for gradient formation taught to biology students for more than two decades. “Seeing is believing” also summarizes the erroneous steps that were needed to elucidate the mechanisms of gradient formation and the path of movement of Bcd. Initially proclaimed as a dogma in 1988 and later incorporated into the SDD model where the broad diffusion of Bcd throughout the embryo was the predominant step leading to gradient formation, the SDD model was irrefutable for more than two decades until first doubts were raised in 2007 regarding the diffusion properties of Bcd associated with the SDD model. This led to re-thinking of the issue and the definition of a new model, termed the ARTS model which could explain most of the physical constraints that were inherently associated with the SDD model. In the ARTS model, gradient formation is mediated by the mRNA which is redistributed along cortical microtubules to form a mRNA gradient which is translated to form the protein gradient. Contrary to the SDD model, there is no Bcd diffusion from the tip. The ARTS model is also compatible with the observed cortical movement of Bcd. I will critically compare the SDD and the ARTS models as well as other models, analyze the major differences, and highlight the path where Bcd is localized during early nuclear cycles.

## History of mechanisms to explain the occurrence of the Bicoid morphogen gradient

### Proclamation of the SDD model as a dogma in the 80’s

**Fig. 1:**
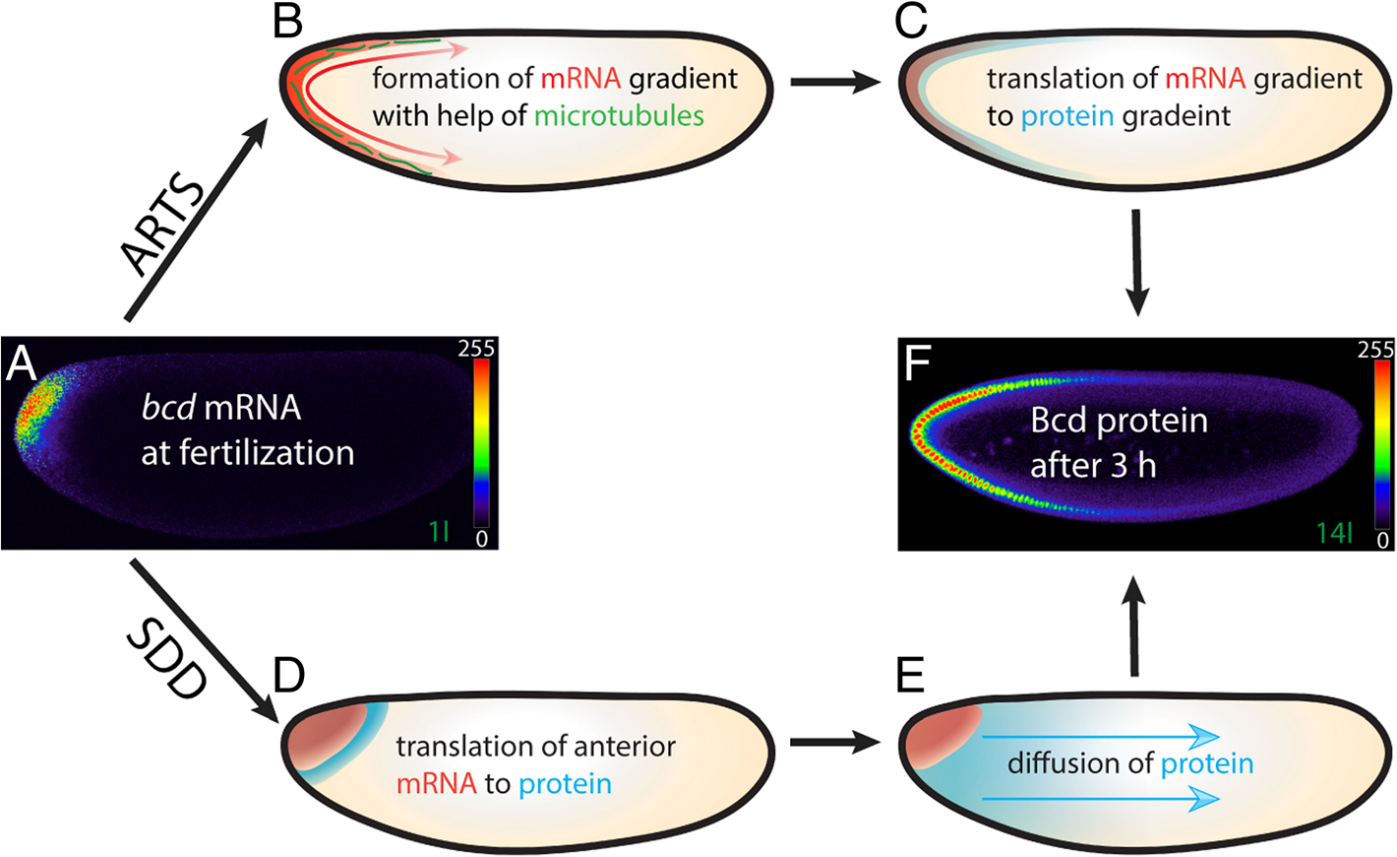
Comparison of Bcd gradient formation, as explained by the ARTS and the SDD models. Pictures represent midsagittal confocal planes or schematic drawings of embryos oriented with their dorsal side up and anterior to the left. Relative intensities of the crude confocal pictures were converted to a color scale with values of 0–255 (8-bit), as shown in inserts of (**a**) and (**f**), respectively. Nomenclature of nuclear cycles follows that of [28]. (**a**) freshly fertilized embryo stained for *bcd* mRNA. (**b**, **c**) in the ARTS (**a**ctive **R**NA **t**ransport, **s**ynthesis) model, the *bcd* mRNA (red, arrows) is actively transported along microtubules (**b**, green) to form the mRNA gradient. The mRNA gradient then serves as template for translation of the Bcd protein to form the protein gradient (**c**, blue). (**d**, **e**) in the SDD (**s**ynthesis, **d**iffusion, **d**egradation) model, the mRNA (**d**, red) is proposed to stay at the tip at all times. The mRNA is translated to produce the Bcd protein (**d**, blue) which diffuses throughout the whole embryo (**e**). After 3 h, the nuclei at the 14th nuclear cycle are filled with Bcd protein which forms a precise morphogen gradient (**f**). Please note that for both models, the start and end points are identical (**a**, **f**), but they differ considerably in their mechanisms

The Bicoid (Bcd) protein and its gradient (Fig. 1f) is one of the fascinating observations in nature. Discovered in the fruit fly, *Drosophila* during the late 80’s [1, 2], it was illustrated in textbooks as a paradigm for morphogen gradient formation. Not only was the morphogen gradient beautiful by appearance, it was also remarkable how the embryo managed to generate an anterior-posterior (A-P) gradient and a coordinate system based on information which initially is stored as a point source as maternal mRNA (Fig. 1a). Within 3 h of development, a precise gradient is generated revealing an exponential decay (Fig. 1f) which strictly follows mathematical rules [3]. *bcd* resides at the top of the hierarchy of segmentation genes [4] to pattern the A-P axis and instructs the gap genes [5] via its concentration gradient from the anterior side. The gap genes, in turn, are expressed in broader domains along the A-P axis and control the so-called pair-rule genes which are usually expressed in 7 stripes [6]. These, in turn, control the segment polarity genes (also referred to segmentation genes) [7] that further subdivide the embryo into smaller units. Finally, homeotic genes are thought the maintain the status of the established segments [8].

The SDD model (S standing for “synthesis”, D for “diffusion” and D for uniform “degradation”) was initially used to explain the establishment of the gradient [2, 9]. The model proposed that the *bcd* mRNA stays strictly at the tip during all developmental stages (Fig. 1d) serving as template for the production of Bcd which itself diffuses throughout the embryo in all directions (Fig. 1e), followed by uniform degradation. The nuclei do not contribute to the shape of the gradient, but rather function as a tool to interpret the gradient [10].

Attempts to validate the model were inconclusive for a long time and it took almost two decades to realize that the diffusion constant of Bcd was far too low to move to the posterior [11] and that SDD model might contain conceptual errors. One of the major drawbacks of the SSD model was its presumption that the Bcd protein diffused throughout the embryo (Fig. 1e). This was fueled by studies where fluorescently labeled dextrane particles injected at the anterior pole were used to simulate the diffusion of Bcd [12]. In retrospect, it was a daring proposal, however, the constraints of this approach were clear from the very beginning. Nevertheless, the approach was too simplistic to assume that a protein would behave like a dextrane particle. Subsequently, other reports measured higher diffusion rates [13–15], calculated to be high enough to explain the SDD diffusion model, corroborated by a recent biophysical model analysis [16].

### The ARTS model as a solution for most physical constraints

In 2009, a new model for Bcd gradient formation was proposed solving most of the physical constraints associated with the apparent slow diffusion constant of Bcd: the ARTS model [17–21], standing for “active RNA transport and synthesis”. The ARTS model incorporates the formation of a *bcd* mRNA gradient and involves active transport of the mRNA by means of microtubules forming a mRNA gradient first (Fig. 1b), followed by synthesis of the protein based on the mRNA gradient (Fig. 1c). One report showed that the mRNA briefly enters the yolk at nuclear cycle 4 [19]. In summary, while the start and end points of the two proposed mechanisms are identical (Fig. 1a, f), it is the mechanism in between where the ARTS model differs completely from that of the SDD model [21].

### Another model

In 2007, a model was proposed to involve Bcd diffusion combined with nucleocytoplasmic shuttling, but no Bcd degradation [22]. The nuclei would serve as reversible traps that affect and slow down Bcd diffusion, while their increase in number with time would counteract the diffusive spread of Bcd. Notably, the Bcd gradient was predicted to be established before the nuclei migrate to the periphery, i. e. before nc 10. Moreover, the gradient was proposed to remain stationary during the remaining 4 nuclear cycles up to nc 14 when cellularization was reached.

### Analysis of Bicoid movement in the early *Drosophila* embryo

**Fig. 2:**
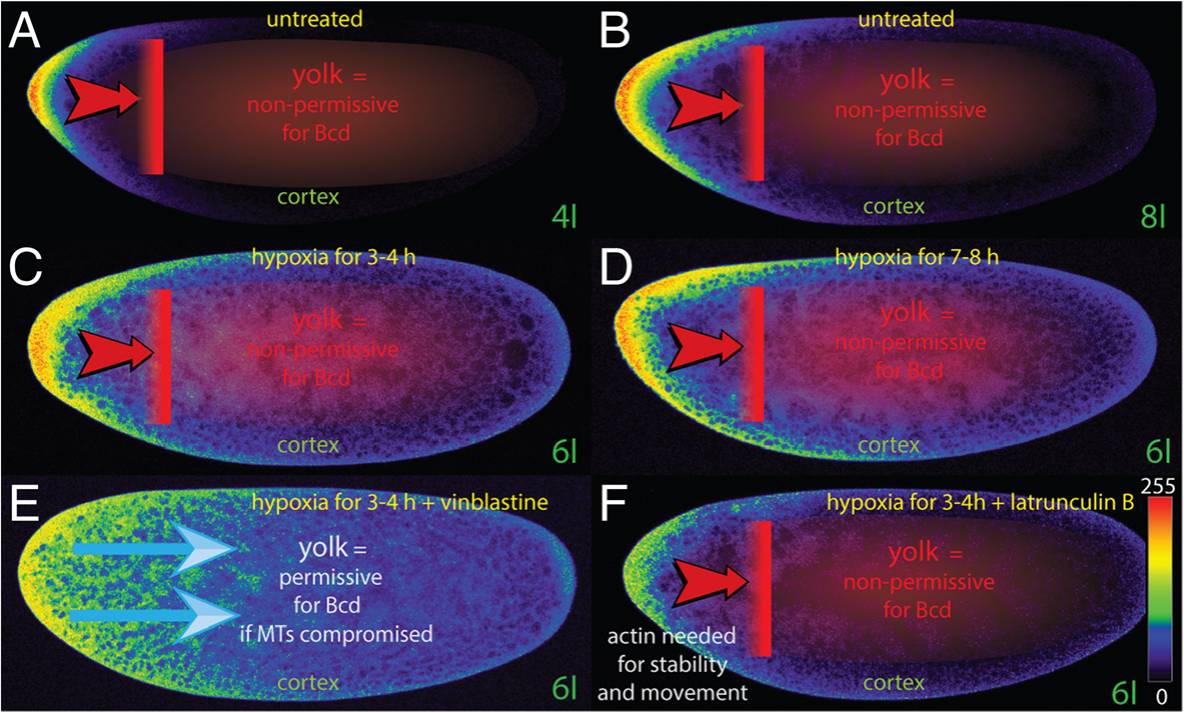
Cortical movement of Bcd and how drugs affect movement and stability of the Bcd protein. Pictures represent midsagittal confocal planes of *bcd*^*+ 5 + 8*^ embryos [20] that produce 3 times more Bcd protein than in wild-type, oriented with their dorsal side up and anterior to the left. Relative intensities of the crude confocal pictures were converted to a color scale with values of 0–255 (8-bit), shown in insert of (**f**). Nomenclature of nuclear cycles (in green) follows that of [28]. Red areas in (**a-d**, **f**) represents the yolk. **a** untreated nuclear cycle (nc) 4 embryo showing the majority of the Bcd protein at the tip and a small gradient is observed. The yolk part (red) serves as a non-permissive territory for Bcd which refutes the SDD model (Fig. 1e). **b** untreated nc 8 embryo showing the Bcd protein moving along the cortex to the posterior. The yolk part (red) serves as a non-permissive territory. (**c**, **d**) nc 6 embryos exposed to hypoxia and “sleeping” [20] for 3 h (**c**) or 7 h (**d**), Bcd still moves along the cortex. Under hypoxic conditions, the yolk (red) still serves as non-permissive territory of Bcd movement. **e** nc 6 embryo exposed to vinblastine affecting microtubular (MT) stability, the yolk becomes permissive and Bcd moves to the posterior in a broad front (blue arrows), as the SDD model would have predicted (Fig. 1e). (**f**) nc 6 embryo exposed to latrunculin B affecting actin structures, the stability of Bcd is strongly affected, as well as posterior movement is slowed down. The yolk (red) still retains its non-permissiveness

To precisely monitor the path of Bcd movement during early development, a sensitive approach was developed that allowed for the study of the spatial Bcd movement during the early nuclear cycles using single confocal sections [20]. This study revealed, for the first time, that Bcd moved at the cortex of the egg but never entered the inner portion filled with yolk (Fig. 2a, b), a feature that could not be observed in the past because live imaging of Bcd was largely impossible due to the elevated background from the yolk. Notably, the inner part of the egg, i. e. the yolk acted as a non-permissive territory. The results from this study immediately refuted the SDD model which predicted that Bcd would move throughout the yolk. If the egg was exposed to hypoxia, i. e. by water submersion, it fell into “sleep” and development was halted. However, development resumed when oxygen was restored. The hypoxia technique permitted the monitoring of the movement of Bcd under prolonged sleeping conditions, i. e. for 3 h (Fig. 2c) or 7 h (Fig. 2d). During this sleeping phase, Bcd still moved slowly at the cortex to the posterior. Again, the speed and location of Bcd movement was not compatible with the diffusion properties claimed by the SDD model [20], nor with that from the nucleocytoplasmic shuttle model [22]. Notably, the inner part of the egg, i. e. the yolk still acted as a non-permissive territory (Fig. 2c, d; [20]. This finding was unexpected, based on the previous knowledge of the diffusion of fluorescent dextrane particles which easily entered the yolk [12]. Data published concurrently by [20] reiterated the notion that the mechanism for Bcd movement was presumably far more complex than previously anticipated. Experiments demonstrated that embryos exposed to smaller drugs directed against major cytoarchitectural proteins such as microtubules (MTs) or actin, also affected Bcd movement (Fig. 2e-f; [20]. Intact MTs appeared to be indispensable for maintaining a non-permissive territory (Fig. 2e). If actin was compromised, Bcd movement as well as its stability was affected leading to a substantially-altered Bcd pattern (Fig. 2f). This data suggested that actin has a dual function for Bcd.

## State of the art

### The SDD model is possible under artificial conditions

The drug treatment data of [20] revealed that the inner part of the egg, i. e. the yolk became permissive for Bcd if embryos were bathed in substances affecting the cytoarchitecture. In vinblastine-treated embryos, the Bcd protein movement behaved exactly as the SDD model would have predicted, i. e. it moved to the posterior in a broad front (Fig. 2e) and the yolk became permissive for Bcd. However, these are artificial conditions since embryos are not exposed to vinblastine in nature. This observation also revealed several apparent weaknesses of the SDD model that were never before discussed. If the model was correct, why should the embryo translate a protein at the tip while the majority remains in the interior of the embryo, never reaching the blastoderm nuclei? Secondly, how would an insect egg three times the size of *Drosophila*, e. g. that of the blow fly *Lucilia sericata* [18, 23] solve the problem of Bcd movement through the yolk where the expansive distance in this large embryo creates further physical constraints? Thirdly, why would nature choose such a difficult path from the tip through the yolk, and then back to the cortex to enter the blastoderm nuclei?

### Why does Bcd not enter the yolk?

Data on the content and structure of the inner yolk is limited, primarily due to microscopic constraints, because the laser of a confocal microscope cannot penetrate deeply into the optically dense yolk layer. Actin microfilaments in the inner yolk were described [24, 25], but require a more detailed description. For MTs, only the spindle apparatus during the nuclear cycles were bright enough to become visible [18]. Further, attempts to stain an internal MT network for visualizing axial expansion and cortical migration had apparently failed [26, 27]. It is possible that the appearance of a MT-network involved in axial expansion and cortical expansion is nuclear cycle-dependent and consequently may be visible only for a fraction of the cycle, as was the case for the cortical MT network transporting the *bcd* mRNA along the cortex [18]. It is plausible to assume that the yolk contains a plethora of cytoskeletal elements that so far have escaped detection. For example, [28] described the existence of long fibrous materials, presumably of MT-origin, that were observed when embryos were squashed under certain salt conditions and the yolk content examined.

### Existence of different Bcd isoforms

Currently, little data is known regarding the alternative splicing of the *bcd* gene resulting in 5 different isoforms, some of which have been characterized [29, 30]. Of these, isoform A [31] represents a small homeobox-less isoform, 149 amino acids (aa) in size which is expressed at vanishingly low levels [30] and therefore was likely undetected by the assay of [20]. The four other isoforms differ in differential use of a splice-acceptor site at exon 3 which is 15 nucleotides apart giving rise to a protein with 5 extra amino acids (+ 5 aa), combined with the alternative use of a translation start point further downstream. The combination of these events allows for the creation of 4 larger Bcd isoforms of 413, 418, 489 and 494 aa, respectively [31]. Interestingly, the + 5 aa form is expressed as strongly as the commonly-used Bcd isoform lacking these 5 amino acids (unpublished data). However, given the prevalence of alternative splicing immediately upstream of the homeodomain, it was striking to learn that this splicing event was not found to occur in a close relative of *D. melanogaster*, *D. pseudoobscura* [32], that gave rise to the − 5 aa isoform only. All studies using a *bcd* cDNA in the past were done based on the original c53.46.6 *bcd* cDNA clone [1] encoding the − 5 aa form. This form allowed rescue of a *bcd*-null phenotype [33], however, it was unknown to what extent any changes of the anlagen under the rescue conditions were corrected, in analogy to the active fate map repair system in *bcd*^*+ 5 + 8*^ embryos that allowed the rescue of embryos with severely altered Even-skipped stripe patterns and thus substantially changed segmental anlagen [20, 34]. A likely scenario could be that the Bcd protein movement as well as its function could be largely dependent on isoforms, a question which has not been addressed. Furthermore, the smallest isoform with 149 aa likely has the capacity to travel faster to the posterior and may be the factor responsible for suppressing pole cell formation as demonstrated in [20]. Consistent with this observation is data that over-expression of the smallest Bcd isoform during oogenesis alters the segmental anlagen in the posterior and concomitantly suppresses pole cell-formation (unpublished data). The smallest Bcd isoform may also be responsible for the surprisingly robust signal resulting from hub activity of Bcd observed at the posterior end [35], despite the fact that only low concentrations of Bcd were measured at the posterior pole [36]. Unfortunately, the experimental setup of [35] did not allow to discriminate between the different Bcd isoforms to give a hint which isoform was actually detected. To resolve the issue of Bcd isoform movement and function, sensitive isoform-specific antibodies would be required, experiments that would be technically challenging, but not impossible.

### Is active transport of Bcd possible?

This question was discussed by [11] in an effort to explain the low diffusion constant of Bcd. An interaction database of FlyBase [31] lists a number of Bcd-interacting proteins such as Bin 1 and Bin 3 [37, 38], Chip [39], Dampened [40], elF4EHP [41] and Fate-shifted [42]. Of these proteins, none were directly associated with a motor protein, thus, any interaction partner may require further proteins linking Bcd to a motor machinery which so far has not been detected. In my view, the strongest argument against active long-range transport of Bcd is the existence of the *bcd* mRNA gradient as the template for the protein gradient [1, 17]. Moreover, the establishment of the mRNA gradient is fast enough to allow for the formation of the Bcd gradient which makes the involvement of active transport of Bcd largely superfluous.

## Conclusions

Why does it make sense to observe Bcd residing and moving along the cortex and not moving towards the interior? Arguably, the most important finding is the cortical location of the mRNA (Fig. 1b) and the definition of the yolk and the cortex as territories which have an impact on the movement of Bcd. From an energy point of view, it would be too costly to establish a path associated with the yolk first which would pose further problems on controlling the movement of Bcd. These are the main reasons why today I believe in what I see.

## Abbreviations

ARTS: Active mRNA transport, synthesis
Bcd: Bicoid
MT: Microtubule
SDD: Synthesis, diffusion, degradation
